# Reports of Societies

**Published:** 1874-08

**Authors:** 


### Reports of Societies.

***Chicago Medical Society.*** *Transactions of the Meeting of June* 15, 1874. Reported by WILL T. MONTGOMERY, M.D.

The Society met in regular session in the parlor of the Gault House, President, Dr. Quine, in the chair. Special order for meeting—Report of Section on Materia Medica.

Dr. Millard read a paper on "Ergot as a Therapeutic Agent." In the discussion which followed, Dr. N. S. Davis said the subject is an important one and may be studied with benefit. It is but a few years since ergot was entirely limited to parturient cases, but it is now known to have a specific action upon the ganglionic nerve centres. Whether it has a specific action upon the vascular system except through the nervous centres he is not now prepared to say. He began to use ergot in hemorrhagic cases as many as fifteen years ago. He used it in combination with iron in those cases, with very satisfactory results. Related a case in which he continued its use for a number of months with decided benefit. Some eminent physicians have recommended the use of ergot in cerebro-spinal congestions. He thinks it is more particularly useful in the aplastic cases. It increases the contractility of the vessels of the brain and cord, and thus lessens the congestion. He had not found it to be useful in sthenic cases.

Dr. E. F. Ingals said he had recently had occasion to look up the literature on the subject of ergot and had come to the conclusion that it does not always have a specific action upon the uterus in parturient cases.

Vice-President Paoli said that forty years ago ergot was known in the drug stores of Europe as a remedy for producing abortions, but pregnant women had repeatedly eaten of the bread made of it without experiencing the specific effect. He does not believe much in its specific action, and does not think much of it as a remedy for anything.

Dr. Pierson had not used it much, and does not think much of it.

Dr. Stilliaus had not had much experience with it in labor cases.

He used it in a case of retained placenta, and produced hour-glass contraction. Had used it in combination with iron in uterine hemorrhage with good results.

Dr. Thompson thinks ergot always acts specifically when a good quality is used. Dr. Walton is of the same opinion.

Dr. Gapin was glad to learn that so many are abandoning the use of ergot, and wants to see a more vigorous stand taken against its use in labor. His idea of its action upon nonstriated muscular fibre was not in accordance with his idea of natural labor-pains. The one was continuous, the other intermittent.

The Secretary, Dr. Hutchinson, declared himself a friend of ergot even if it is abused. From his own observations he has no doubt that it produces uterine contractions. He had used it in connection with other remedies in hemorrhages and cerebro-spinal troubles, when it seemed to do good. He is careful in giving it in labor, but thinks, if properly administered, it is a safe and useful remedy, and should continue to use it.

Dr. Etheridge being present, Dr. Earle wished to hear from him upon the action of ergotine. Dr. Etheridge said that ergotine is being extensively used hypodermically in the treatment of uterine fibroids, and with good results. A number of cases have been reported as cured. It acts through the vaso-motor nerve centres, shutting off the supply of blood to the tumor. He thinks the action of ergot has been clearly demonstrated by the experiments for the cure of epilepsy. He referred to quinine as an oxytocic, and related a case in which it seemed to have this effect. A lady, seven months pregnant, was having chills and he prescribed quinine in gr. iv doses, and when he called next day, found she had miscarried. There was no sign of it before the medicine was given.

Dr. Earle referred to a paper on ergotine in the treatment of uterine fibroids, by Dr. Parvin, published in the *American Practitioner*. The hypodermic injection should be used once a day, and was first made in the region of the uterus, but it is found to act quite as well given in any other part of the body.

Dr. C. M. Fitch has discontinued the use of ergot in cases of labor, but has used it with apparent benefit in hæmoptysis. He has had no post-partum hemorrhage for a long time, and thinks it is because he has used stimulants and paid close attention to keeping the extremities of his patients warm in parturient cases.

The President, Dr. Quine, asked a question as to the distribution of nerves to the uterus and their physiological action.

Dr. Thompson said she could not answer directly, but had observed cases of labor in which the neck of the womb was flaccid, while at the same time there was firm contraction of the body of the organ.

The President further inquired if any one had had any experience with ergot in cases of threatened abortions.

Dr. Fitch had used it in one case, and the abortion was prevented and the case went on to full term.

Dr. Montgomery said he was called to a case of threatened abortion at the end of the third month, and on making an examination found the membranes protruding from the os, and decided that the case was too far gone to arrest. As there was considerable hemorrhage, gave ergot to hasten the expulsion of the fœtus, but it was not expelled while the patient was under observation, about a week, though the hemorrhage ceased and the patient made a rapid recovery. The case was lost sight of, so that he did not know whether she went on to term or not.

Dr. Hutchinson said it was another example of how patients would sometimes recover in spite of the doctor and his medicines.

Dr. Quine had given ergot in one case, but it did not arrest it. He has no doubt of the specific action of the drug, and thinks it is as reliable in its action as neurotic remedy is in its action. The dose modifies its action; small doses producing natural contractions, large doses continuous. When given in moderate doses it produces increased vascular tension. In large doses it first increases vascular tension, but continued relaxes it. He had produced dilatation of the os by large doses, but contraction by small doses.

After some explanations the discussion closed, and after miscellaneous business was disposed of, the Society adjourned.

*Meeting of July* 6, 1874.

Society met as usual in the parlor of the Gault House, President, Dr. Quine, in the chair.

Order of meeting, reports of cases, and presentation of pathological specimens.

The Secretary being absent, Dr. Graham was elected *pro tem.*

Dr. Strong related the following case, and asked for information as to diagnosis and treatment. The patient, a young lady, 19 years old, always had good health until about one year ago, when she began to feel a stiffness in the calves of her legs. She was attending school at that time, and had to walk considerably, and at first thought the trouble arose from this, but her knees soon began to get stiff, and her legs to draw up, so that in a short time she was unable to walk. Dr. Strong first saw her about four months ago. He found the patient large and fleshy, but her flesh was extremely soft and flabby. Her legs were firmly flexed upon the thighs, but there was no bony anchylosis of the knees, and by persistent effort he was able to extend the legs to an angle of about 45°. He was not able to find the tendons of the hamstring muscles, but found a firm cord in the centre of the popliteal space, which appeared to be the tendons united. There was considerable œdema of the feet and legs, but he thought it was due to the dependent position. Sensibility perfect. The patient has not had any pain, and he was not able to detect any tenderness along the spine. She eats and sleeps well, and says she feels well every other way. Began to menstruate at 16, and was regular until two months before he saw her, since which time she has not had her turns. The Dr. ordered tonic medicines, stimulating liniment, and passive motion, but had found no improvement.

Dr. Mary Thompson suggested pelvic cellulitis as the probable cause of the trouble.

The President thought it more probably depended upon some lesion of the lower portion of the spinal cord.

Dr. Strong thought if the trouble had come from cellulitis the patient would have had pain.

The President referred to a case of cellulitis in which there was very little pain.

Dr. Stilliaus reported the case of a young lady 21 years old, who had been sick since she first began to menstruate seven years ago. She had been under treatment most of this time, but he did not see her until ten weeks ago. He found her apparently well nourished, but complaining of pain and stiffness of one knee, and general hyperæsthesia of the integumentary surface

She was vomiting a good deal; was only able to retain pie and milk. He prescribed anti-emetics, and put extension upon the limb, but not finding any improvement from this treatment, three weeks ago began the use of tonics and electricity. The vomiting has ceased, and the patient is able to walk, but has had a hacking cough for the last four days, and still says she don't eat any. The Dr. said he did not know what her trouble was unless it was hysterical.

Vice-President Paoli thought the case hysterical, and said he had found the tinct. asafœtida and tinct. valerian to act well in such cases.

Dr. E. F. Ingals reported a case of epileptiform neuralgia, with tonic, muscular spasms. He was called about midnight, June 24, to see the patient, Miss B—, about 20 years of age. The messenger informed him she had been suffering from severe cramps of the arms, legs and stomach for three or four hours. He found the patient in bed, apparently suffering but little, and was told she had improved much within an hour. Upon inquiry he learned that she had been suffering from occipital neuralgia of a not very severe type, for several days, the pain commencing late in the afternoon, and continuing three or four hours. The patient stated that during the previous afternoon the pain came on more severe than usual, and was shortly followed by chilly sensations of the feet and legs. These were soon succeeded by cramps of the same parts, then of the hands, and finally of the stomach. Upon examination found the skin cool and moist, pulse slightly accelerated, pupils normal, tongue slightly coated. No vomiting or purging, but bowels open. Some pain, especially in abdomen. The pains had been paroxysmal. Prescribed morph. sulph., gr. $\frac{1}{4}$ and chloral hydrat, gr. x, to be repeated once in three hours if the convulsions returned. The next day he found her suffering only from moderate headache and soreness of the muscles which had been convulsed. The spasms did not return during the night, but the patient slept little. About a year ago he treated the same patient for obstinate intermittent neuralgia. She lived at the time in a damp basement, with poor sewerage, from which she was finally induced to remove, though not until the neuralgia had yielded to treatment. At that time quinine and iron utterly failed to give relief, but the patient promptly recovered upon the use of granules of strychniæ sulph., gr. $\frac{1}{16}$ each, given three or four times daily. Subsequently she enjoyed good health until her present illness. Careful inquiry revealed the fact that the old attack had been preceded by tonic convulsions similar to those of the present sickness. Remembering the former treatment, he at this second visit, notwithstanding the convulsions, prescribed strychnia sulph. in granules of gr. $\frac{1}{16}$ each, to be taken four times daily. That evening, about five o'clock, convulsions of the extremities and maxillary muscles again occurred, and lasted about three hours. He called at five P. M., the following day, and found the patient lying on the lounge, feeling very well—no headache, no pain, and a little appetite. He congratulated himself that convalescence had been established, but about nine o'clock of the same evening, a messenger informed him that spasms had again set in about half an hour after his visit, and were still very severe. He found the patient still in the convulsion so far as the hands were concerned, but spasm of other parts had given way. The fingers were firmly flexed by tonic muscular contraction. Inspecting the medicine, he found the druggist had made pills instead of furnishing the granules ordered. Discontinued the strychnia, and ordered morph. and chloral, as before. The following afternoon (27th inst.) ordered zinci valerianas, gr. ss. at a dose, made into pill, with confec. rosae, to be given three times daily. Convulsions, milder than before, occurred that evening. Two days later, he found the patient comfortable, and that she had escaped pains and cramps the previous evening. What was the cause of this sudden cessation of neuralgic pains, and the accompanying convulsions? The valerianate of zinc will be the answer, he thought, of nine out of ten physicians; but not so, for he found that the patient had not taken the medicine, owing to a misunderstanding on her part, about the nature of the pills. The medicine was begun, but not early enough to gain any credit in the case. Two days later, he found her still improving. She had suffered no more cramp, and now wanted to go to the country on a visit of several days. He ordered the medicine continued for a day or two, in doses of gr. j, to be followed by a ferruginous tonic.

The Dr. remarked that the case seemed interesting, in the first place, on account of the convulsions attending an otherwise simple case of intermittent neuralgia. The first question occurring to us is, what caused the convulsions? Doubtless the same cause operating upon the cerebro-spinal axis produced convulsions, which had formerly caused occipital and abdominal neuralgia; but the exact nature of this cause he was unable to state. The patient was neither of a rheumatic or gouty diathesis. She had not been exposed to lead poisoning, and could not be properly called anemic. The pains were distinctly intermittent, and so we might suspect malaria, but when we remember that many nervous affections not dependent upon malaria exhibit an intermittent character, we are still left in doubt. He said it seemed to him as unphilosophical to call every intermittent affection malarious as to commit the common blunder of calling every disease rheumatism, or syphilis, from which the patient recovers while taking iodide of potassium.

Secondly, he wished to call attention to the use of strychnia sulph. in doses of gr. $\frac{1}{16}$. Whether in this instance the druggist was accurate in his preparation, is a matter of doubt. He believes ordinary drug clerks are hardly competent to dispense such active medicines in pills or powders. Therefore when granules prepared by experienced pharmaceutists cannot be obtained, strychnia should be given in solution, notwithstanding its intense bitterness. With regard to the dose, standard authors vary from $\frac{1}{32}$ to $\frac{1}{8}$ of a grain, but they are not always safe to follow. He believed gr. $\frac{1}{16}$ to be too large a dose to begin with, and should not have given it in this case had he not known the patient's previous history, even though he had himself taken gr. $\frac{1}{10}$ three times daily without feeling it. He had in mind a patient suffering from hemiplegia, who for several weeks took about gr. $\frac{1}{20}$ three times a day, but finally had severe convulsions, which immediately subsided when the medicine was suspended. His preceptor once saw a lady who evidently died from the effects of strychnia administered in gr. $\frac{1}{20}$ doses three or four times daily for several days. He believes this remedy possesses a cumulative action. That is, while a given dose may be taken for a considerable time without ill effects, exactly the same dose—and by this he did not mean the same dose from the bottom of a bottle containing a solution of strychnia, which might be much stronger than that taken from the top, but the same dose of the medicine itself—may suddenly give rise to its toxical effects.

His experience with the valerianate of zinc in this case was purely homœopathic, but to him another caution against jumping at conclusions with regard to the action of medicines. If the patient had taken the medicine before the convulsions ceased, the one out of ten who presumed to doubt its effects would at least have been thought presumptuous.

Dr. C. M. Fitch thought a patient who had once taken an overdose of strychnia remained more susceptible to it for a long time. He gave a patient who had once had an overdose, gr. $\frac{1}{60}$ three times a day, and, after a few doses, got the poisonous effect.—President had not been a believer in the cumulative action of medicines, and thought the trouble, in most cases, was brought about by giving the medicines faster than they are eliminated, and thought this was true of strychnia. He agreed with Dr. Fitch in reference to increased susceptibility.—Dr. Pierson gave strychnia to a patient, who became scared, and presented symptoms of poisoning, but when he gave it in the same dose, disguised, it had no bad effect.—Dr. Strong once gave a patient with Bright's disease two doses of strychnia, gr. $\frac{1}{32}$ each, and the last dose was soon followed by convulsions simulating those produced by it, and the patient died next day. Was not sure whether the convulsions came from the medicine or uremia.—Dr. Taggart had often prescribed strychnia in gr. $\frac{1}{16}$ doses, but had not seen any bad effect from it.—Dr. Earle had recently seen a patient, a hard drinker, who, with suicidal intent, took 400 grs. of chloral hydrat at once, without the desired effect. The following night he took what was purchased for gr. x of morph. sulph., but made another failure. The patient had not been addicted to the use of opium, but as the chloral did not kill him, the Dr. thought he had possibly taken gr. x of morph. too.—Dr. Knox once prescribed 480 grs. of chloral in solution for a case of delirium tremens, and the patient took it all in one night, without any apparent effect. He thought the alcohol antagonized its action.

Dr. Earle reported a case of post-pharyngeal abscess, a sequela of scarlet fever. As this case will soon be published in full, we omit an abstract of it here.

Dr. Fitch presented a specimen of polypoid tumor of the uterus which he had removed from the patient of another physician by means of the galvano cautery. The tumor began to appear about one year ago, and the patient had since suffered from excessive hemorrhage, at times almost fatal. The tumor was attached high up in the canal of the cervix by means of a short, thick pedicle, and, at the time of its removal, was nearly as large as a goose's egg, and of a dark red color. The loop of wire was passed around the pedicle of the tumor, and heated by means of nine large-sized Bunsen cells. The incision was as smooth as if it had been made with a knife. No hemorrhage followed, and the patient went on to a rapid recovery.

After some discussion as to the nature of the tumor, the Society adjourned.

---

***Chicago Society of Physicians and Surgeons.*** *Transactions at Regular Meeting, June* 8, 1874. Reported by RALPH E. STARKWEATHER, M.D.

The Society met as usual in the parlor of the Grand Pacific Hotel, the President, Dr. John Bartlett, in the chair.

The following were elected to membership: Doctors M. O. Heydock, H. M. Bannister, H. W. Jones, and D. K. Steele.

The names of Doctors H. A. Johnson and H. K. Newton were proposed and referred.

Dr. Hyde read a paper, "Notes on the Microscopical Appearances of the Brains of the Insane," prepared by Dr. Walter Kempster, of the Northern Asylum for the Insane, at Oshkosh, Wisconsin, formerly of the New York State Lunatic Asylum, Utica. He had made microscopical examinations in forty-nine cases. Numerous slides were exhibited of sections, made mostly through the third left anterior convolution, illustrating the lesions of acute mania, the large sclerous patches in chronic mania, and the dementia of syphilitic paralysis. Numerous micro-photographs were likewise shown, illustrating the lesions of cerebro-spinal meningitis; of numerous colloid masses in the medulal oblongata, and large degenerated masses with dense fibrous investing membrane in the spinal cord, opposite second cervical vertebra, each illustrative of acute mania. The student is met with the stereotyped phrase that there are no lesions discoverable peculiar to insanity. For a number of years Dr. Kempster has been making systematic microscopical study of the brain, examining the lesions of all forms of insanity, from acute mania to dementia, including puerperal and epileptic insanity. In each and every form he has found a marked lesion, so that certain lesions may be grouped together as common to certain forms of insanity, to which lesions any particular type of insanity may be palpably due. Six different forms of degenerations and lesions were elaborately described.

A recess was then taken, during which the Society examined the specimens and micro-photographs.

On motion, a vote of thanks to Dr. Kempster was passed, and he was elected an honorary member of the Society.

The amendment to the constitution offered at the last meeting by Dr. Jackson, was taken up and adopted.

*Transactions at Regular Meeting, June* 22, 1874.

The Society met at the Grand Pacific Hotel, the President, Dr. John Bartlett, in the Chair.

The members were desired by the President to accept the intimation that the meetings would be called to order, promptly, at eight o'clock. Owing to the absence of Dr. Hyde, Dr. Wood was elected Secretary *pro tem.*

Dr. H. A. Johnson received an unaminous election to the membership of the Society.

The following By-Law was, upon motion of Dr. Hay, adopted:

The privilege of nominating new members to the Society is restricted to the nominating, by each member, of one name, each year.

Dr. Trimble read a paper on Eucalyptus, the substance of his remarks, as he says, being chiefly collected from various articles written upon the subject within the last year or two, in periodical journals; the doctor stating that he had no practical knowledge of the effects of this medicine.

Dr. Wood.—It seems important to remark that when eucalyptus is used in intermittent fever, if it is to prove useful at all, one dose generally accomplishes the desired effect; the fever does not return.

Dr. P. S. Hayes.—The tincture is made stronger in alcohol than the ordinary tinctures; hence, when used with other tinctures it precipitates; it disguises the taste of quinine.

Dr. P. S. Hayes reported a case of multilocular sero-cystic ovarian tumor. Operation, electro-puncture. Recovery. No abstract of this case is made, as it will soon be published in one of the medical journals.

Dr. Etheridge presented a report of a case of corroding ulcer of the uterus. This is a comparatively rare form of ulcer, and its diagnosis in this case is confirmed by the opinions of Drs. De Laskie Miller, Byford, and T. G. Thomas, of New York.

Patient first came under notice July, 1871, and was fifty-nine years of age. She passed the turn of life quite suddenly, in 1860. During the first twelve years after marriage she suffered from menorrhagia, at times so profusely as to prove nearly fatal. This almost wholly disappeared upon bearing children, of whom she had six. In many years of the period of child-bearing, she had *stomatitis materna* severely.

After having had at wide intervals, three hemorrhages, Dr. Etheridge was called to attend the case. No examination being allowed, he ordered a vaginal injection of tannic acid—forty grains to the fluid ounce—to be used as often as necessary. Relief was afforded for four months, but the shock of the great fire in this city renewed the bleeding; the discharges, however, were not offensive. The only pain was a back-ache, a hot, burning pain—not lancinating.

Upon examination per vaginam, the cervix uteri was found half eaten off, the edges of the ulcer being ragged—projecting unevenly; its surface bled upon the slightest touch. Beneath the site of the os, there was an indurated nodular ring, around the vagina, through which the speculum always slipped with a sort of snap or jerk. Uterus movable; pelvic glands and viscera normal. A solution of the pernitrate of iron temporarily checked the hæmorrhagia, and afforded the longest interval of freedom from hemorrhage. Other styptics were used—carbolic acid and tannin, and chromic acid; injections of the latter produced pain, often vomiting, but checked the bleeding. Fifteen grains of the chromic acid, in a tea-cup of warm water, seemed to be the minimum strength that was efficacious in this case. There were, at length, three months of freedom from hemorrhage. Gradually, there came on vesical trouble, and a distressing feeling in the stomach, followed by bleeding. The tissues contiguous to the ulcer now became infiltrated and indurated, probably lessening hemorrhage; the vagina was well nigh filled with these growths; there was the usual cancerous cachexia. Morphine was used to allay pain, the latter weeks of life. No necropsy allowed. Remedies for constitutional treatment were used, such as arsenious acid, red clover, iodine, cundurango. Prof. Schiff's treatment of uterine cancer was tried—pancreatine solution, with the view of dissolving the cell of the ulcer, with no satisfactory result attributable to pancreatine.

Very particular attention was called to a point in the history above given, not alluded to in books or lectures—*the nursing sore mouth of the mother* during lactation, who, *later in life*, *suffered also from malignant disease.*

The cases of two other women were cited, who died after the menapause from malignant disease, having in former years suffered from nursing sore mouth. *Is there any connection or dependence between these two conditions?* If there be, what course ought the physician to pursue, with the view of preventing the development of malignancy in later years of life?

Dr. Wood stated that he knew of a family of three sisters, all of whom have had stomatitis. Two of them, in later life, died of internal cancer; the third is now sick with the same disease.

Dr. Hay suggested that a table of statistics could be written, with the inquiry made by Dr. Etheridge kept in view; and it would be important if such a relation between stomatitis materna and forms of malignant disease could be established.

Dr. Etheridge had spoken to many of the older physicians who had given many instances similar to that of his case. The point is, whether this relationship can be established in all or sufficiently numerous cases.

Dr. Trimble reported a case of epilepsy in a boy of four years of age, probably due to injuries received twenty months previously, by a railroad accident. Of late, the seizures had increased in frequency, severity and mental disturbance. To eliminate the idea of worms being an exciting cause, appropriate treatment was adopted, and a few expelled. The potassium bromide was given in four grain doses, three times a day; but there were still as many as twenty convulsive attacks daily. Belledonna was given with the bromide. The cerebral condition soon became such, that despite the usual practice and opinion I felt obliged to bleed, and placed four large leeches on the temples, drawing at least six fluid ounces of blood. The dose of the bromide was increased to eight grains; iron and bark were also given. The patient had no more seizures after the third week from the date of bleeding.

Dr. F. H. Davis, in behalf of Dr. Andrews, presented nine renal calculi, taken from one patient by Dr. Andrews.

Dr. Walter Hay, in behalf of Dr. Ben. C. Miller, exhibited a catheter which he had devised for the purpose of making medicated applications to the urethra and other tracts. It was a simple silver catheter, with a stylet having a bulbous point, just below which a piece of medicated sponge was folded. In the stylet, near the handle, a thread was cut, upon which revolved a disc; the stylet could thus be pushed forward out of the sound to the desired distance and object of treatment, or the sound withdrawn upon the stylet, and the medicated sponge, lying next the bulbous point, could be applied wherever desired.

Upon motion, the Society adjourned.

*Transactions at Regular Meeting, July* 13, 1874.

The Society met as usual, in one of the parlors of the Grand Pacific Hotel, Dr. Jno. E. Owens, Vice-President, in the chair.

Dr. A. K. Norton was unanimously elected a member of the Society.

The notice of the paper read at the last meeting by Dr. P. S. Hayes on " Multilocular Sero-cystic Ovarian Tumor," should have stated that the operation of electro-puncture resulted in complete recovery.

Dr. Hyde read a report of a case of variola, modified either by antiseptic treatment or previous protection, of which the following is a brief summary:

The patient, a boy of five years of age, was seen by Dr. Hyde the second day of his illness, and had high fever, pulse, 130, temperature, 105.5° F.; constipation, coated tongue, sleeplessness and jactitation. The cheeks were suffused with a damask stain, with well-defined limits on every side; no redness of the fauces, sore throat or coryza. The boy had been vaccinated in early infancy, but upon inspection of the arm I discovered a single punctate cicatrix, such as might have resulted from a wound of the arm by a shoemaker's awl.

On the fourth day a complete defervescence occurred. A general eruption then appeared, gradually extending and invading the palms of the hands, the soles of the feet, and the pharynx; rapidly passed through the stages of pimple and vesicle, until a well defined umbilication occurred, vesicles generally discrete; the odor of small-pox was perceptible.

Some two years ago the attention of the profession was attracted to a form of treatment by antiseptic solution, first reported in a medical periodical of Canada, and largely copied by home journals. It consisted of carbolic acid, dr. j; of Squibb's pure medicinal sulphite of soda, dr. x; and f. oz. vj of water. Dose, children, one-half to one drachm; to adults, a tablespoonful, every three hours. Externally, a solution of carbolic acid (dr. ij), to glycerine (oz. iij). A febrifuge was also advised, of potassa chlorate, spts. nitre, and liq. ammonia.

Dr. Hyde employed the above treatment, modified, and on the sixth day of this patient's disease ordered: R. Acidi carbolici cryst., dr. j; sodæ sulphitis (Squibb's pure medicinal), dr. x; aq. menth. piperitæ, aq. puræ, *aa* f. oz. iij. M. Sig. one teaspoonful every three hours, day and night.

The external lotion was made of less strength, and modified thus: R. Acidi carboli, dr. j; glycerine, f. oz. iv. M. and use on the exposed parts of face and neck.

The result was as gratifying as surprising. Seventy-two hours afterwards, the ninth day of the disease, the little patient seemed practically cured of his malady; the eruption had everywhere subsided; no intumescence of the skin between the vari; the itching of the skin was very slight, and the child was dressed and at play. The subsequent history of the case is that of perfect restoration to health.

Dr. Hyde then alluded to the eight varieties of varioloid spoken of by Dr. Aitken, contrasting the present case with them, and developed several interesting points and deductions, and, in conclusion, said that there is as much evidence to show that the sulphite of soda is capable of controlling variola as there is in favor of the remedial influence of the transfusion of blood; it should be carefully and faithfully tried in each case.

In remarking upon Dr. Hyde's use of the sulphite of soda, Dr. Chapman said that two years ago, at Ann Arbor, they had quite a siege of small-pox in the college, during which the patients were put under treatment by the sulphites with great success. Those treated as soon as the attack became apparent were greatly benefited; the disease was modified in severity, there was no eruption on the palms of the hands or the soles of the feet, though the patients had never been vaccinated. The sulphite of soda and the sulphite of magnesia were used, a drachm of either salt in a glass of water, drinking of the same from time to time. A strong lotion of carbolic acid was used. The pitting was severe in two cases only.

Dr. F. H. Davis.—Was carbolic acid exhibited internally?

Dr. Chapman.—It was not; but a carbolic acid gargle was used in some of the worst cases.

Dr. F. H. Davis.—I had supposed that the use of the carbolic acid internally had as much to do in contributing to successful results and relief as the sulphites. I have tried the sulphites in three or four cases, and none died, though two cases were of the confluent variola. The attack did not seem to be aborted; there was some pitting in the confluent; used no carbolic acid externally.

By the President.—It is to Dr. A. Fisher the profession is indebted for establishing the dose of the sulphites. It is usually given in too small quantities, at too infrequent intervals. Dr. Blake, in 1864, reported to the American Medical Association a case of pyemia with pneumonia, pleurisy and endocarditis, with comp. com. fracture of the tibia. He had ordered drachm doses every four hours: at the end of thirty-six hours the patient had recovered from the pyemia; it was discovered, however, that four drachm doses of the lime sulphite had been given instead of drachm doses. The medicine should be given some time, to prevent relapses.

Dr. Fisher.—I have frequently given one ounce of the soda sulphite in twenty-four hours: even an ounce and one-half. The ordinary lime sulphite of the shops is totally unfit for use: that made by Dr. Squibb is the most suitable. In diarrhœa and cholera, a half drachm of lime sulphite may be given every four hours; have never seen bad effects in the use of the sulphites, and think I have aborted many cases which otherwise might have been fatal. I give it in every disease of a septic nature. In one case there was an infant in whom vaccination did not take. I gave the sulphites, and no eruption followed. Sulphite of soda may be given with peppermint water, in an emulsion; it is not distasteful to children. The soda is preferable in case of constipation. The sulphites are of no benefit in inflammatory diseases.

Dr. Andrews thought the sulphites were given too timidly. You must saturate the patient with the drug. Small doses are not so efficient as large doses.

Dr. Andrews then addressed the Society in partial review of the Report of the Supervising Surgeon of the U. S. Marine Hospital Service, comparing the same with a study of the cases in hospitals of London and Paris.

The only subject alluded to was that of the diseases of two races—the Teutonic and Negro, of this country; and the Teutonic (English) and Gallic race (French) of Europe; and also of their different physiognomy and facial angle, and measurements. His conclusions, in brief, were that the British Teuton, as compared with the Gallic Parisian, is prone to alcoholic disease:

the latter to venereal diseases. Some attribute the difference to influence of climate and food.

In this country we have the two races side by side, and in the same relation as to climate and food: say for the past three hundred years they have grown side by side.

In the Marine Hospital Report, the colored sailors, by a large percentage, are under treatment for venereal diseases, while of the whites much the larger percentage is in for delirium tremens.

Dr. Hay.—There is one view Dr. Andrews has omitted to take which a close observation of the negro race for twenty-seven years has led me to adopt. In the Teuton, the tendency to drink is partly hereditary; the negro in service has been kept sober, regular and steady in habits, and probably his freedom from alcoholism is due to this exemption from hereditary alcoholism. I mean the transmission of certain modifications of the nervous system, due to the continued use of alcoholics, by successive generations.

Dr. Andrews did not doubt this, but had never been able to satisfy himself as to the degree of exemption.

The President read a deferred paper on a means of facilitating the introduction of Barnes' dilators. The devices of Skene and Bishop were referred to, and the means suggested were offered as additional to these.

Dr. Bartlett's plan consisted in the rolling up of the ordinary dilator about the supporting rod or tube, and the maintenance of it in that state of compactness, till the introduction was effected, by means of a cord or tape, adjusted about it in such a manner as permitted of the ready release of the bag from the coils of the string. The dilator having been exhausted of air by suction with the mouth, is compactly rolled up on its long axis, and secured in this state by a temporary string at each end. A piece of wire, as a small stylet from a catheter, is then laid parallel with the bag and its supporting staff. The wire, bag and staff are then lashed together by what Dr. Bartlett called a key-wire stitch. The cord is first tied around the wire, at a point corresponding to the tube end of the dilator, it is then passed under, and then over the bag and staff, and then placed under the wire, and looped over it, the direction of the string being thus reversed. The cord is now carried under and over bag and staff, in a direction contrary to that of the first stitch, and is again carried under the wire and looped over it as before. This process of binding the bag on to the staff by looping the string over the wire, is continued till the dilator is lashed to the staff in nearly its whole extent. The stitch is ended off by taking several looped turns over the extremity of the wire, this latter is then withdrawn, so that its end rests concealed just below the presenting extremity of the bag The temporary strings are removed as the stitch is being made, or afterward. The dilator being introduced, the string is discharged from it by withdrawing the key-wire. Any form of cord will answer the purpose, but very narrow tape, oiled, is the most suitable; the lashing should not be too tight. Dr. B. thought it probable that this stitch might be usefully employed for other obstetrical or surgical purposes.

The meeting closed with the presentation of a testimonial by Dr. Hay, in behalf of many members of the Society, to Dr. Hyde, the Secretary, of a set of Gouley's Sounds, and a Mott's Speculum, in recognition of that officer's long and faithful services to the Society.

---

***Æsculapian Society of the Wabash Valley.*** *Proceedings of the 29th Semi-Annual Meeting.* Reported by G. T. RAGAN, Secretary, Neoga, Ill.

By invitation of the Clark County Medical Society, the Æsculapian Society met in Marshall, Ill., May 27th, 1874.

At 10 A. M. the meeting was called to order by the President, Dr. J. D. Mitchell, of Terre Haute, Ind. By request of the President, Rev. E. M. Pilcher offered prayer.

The minutes of the last annual meeting, held at Paris, Ill., were read by the Secretary, and approved.

Sixteen members were present, viz: W. M. Chambers, Charleston, Ill.; W. S. Goodell, Effingham, Ill.; N. S. Freeman, Campbell, Ill.; J. L. Hays, Paris, Ill.; Wm. Massie, Grandview, Ill.; J. D. Mitchell, Terre Haute, Ind.; D. O. McCord, York, Ill.; A. J. Muller, Paris, Ill.; W. S. Martin, Tuscola, Ill.; J. M. McKown, Arcola, Ill.; G. T. Ragan, Neoga, Ill.; M. Rowe, Dudley, Ill.; J. M. Steele, Grandview, Ill.; John Tenbrook, Paris, Ill.; J. Washburn, Tuscola, Ill.; L. J. Willien, Terre Haute, Ind.; and during the session eleven new names were added to the roll, after presentation in due form and an examination by the Board of Censors, viz: O. C. Tobey, Westfield, Ill.; A. K. Mosely, Grandview, Ill.; R. C. Prewitt, Marshall, Ill.; S. F. Storer, Darwin, Ill.; R. F. Williams, Casey, Ill.; Simon Jumper, Darwin, Ill.; W. H. McNary, Martinsville, Ill.; D. C. Nicason, Melrose, Ill.; A. T. Steele, Ashmore, Ill.; Jas. M. Barlow, Westfield, Ill.; R. C. Bradley, Marshall, Ill.; making, in all, a membership of sixty-two.

An address was delivered by Rev. Mr. Pilcher in behalf of the Clark County Society and citizens of Marshall, welcoming the Society to the hospitalities of their city. This address was responded to, in behalf of the Æsculapian Society, by Dr. William Massie, of Grandview, Ill., in his peculiarly happy manner.

On motion of Dr. McKown, the following members were appointed a committee to report resolutions expressing the feelings of the Society in reference to the death of Dr. F. R. Payne, viz: Drs. Chambers, Tenbrook and McCord.

After the usual preliminary business and dinner, the Society listened to a paper on "Cerebro-Spinal Meningitis," by Dr. L. J. Willien, of Terre Haute, Ind. On motion, the paper was received, and ordered to be filed. The following is a synopsis of this paper:

On the uncertainty of the epidemy of the said disease, since it is often mistaken for other diseases in malarial districts, having cerebral or cerebro-spinal complications. The disease has two well-marked forms, malignant and sporadic; the former ending fatally, and the latter being, most of the time, curable. That the disease is of an inflammatory nature, with serous exudation, is shown by post-mortems. Symptoms vary in gravity according to the idiosyncrasy of the patient and complications with other diseases. The main object of the practitioner is to understand fully the symptoms of cerebro-spinal meningitis and the diseases with which it is apt to be complicated.

A table of differential diagnosis is then given between the malignant epidemic and sporadic forms. The treatment indicates no special remedies, with the exception of the use of quinine, belladona, ergot, bromide potassium, and calabar bean, these remedies having undoubtedly given most satisfactory results.

The paper is closed with a report of a genuine case of cerebro-spinal meningitis, sporadic form, terminating favorably.

Dr. D. O. McCord has had a great many cases on the Wabash; considers them inflammatory in character, uses large purgatives, calomel and ipecac, followed by tonics. If he could get reaction, could save his patients.

Dr. Mitchell has always considered it very difficult to make a diagnosis, or, rather, all the true conditions are not met with in any case. Takes the ground that it is a malarial disease—rheumatic. Many of the attacks are similar to our malarial or congestive fevers. Most of the cases had contraction of the muscles. Some years ago had quite an epidemic of this disease. All died but one, which was treated by ten grain doses of quinine. Would use active emetico cathartics at first, but no purging afterwards.

The disease is not inflammatory in its inception, but may become so afterwards. The recent epidemic was very violent in form, and not the same disease as spotted fever.

Dr. J. L. Hays, of Paris, Ill., Chairman of the Committee on Surgery, made a report of cases in his own practice.

1ST CASE—Organic stricture of urethra in a male; relieved by operation.

2ND CASE—Obstruction of the trachea by coffee grain; successfully relieved by tracheotomy.

3RD CASE—Cataract of right eye, with chronic irido-choroiditis, supervening on an operation previously made for cataract on left eye. The operation of enucleation of the left eye, with a subsequent operation for cataract on the right eye, succeeded in opening the eye of the blind.

4TH CASE—Extirpation of the eye-ball.

In connection with this report was a case of acute necrosis of the tibia, reported by the Secretary, with natural expulsion of the bone entire.

The report was discussed by Drs. Chambers, Willien, McKown and Miller. Dr. Hays remarked a difference in periostitis and erysipelas in diagnosis. Erysipelas is always preceded by a chill.

Dr. Willien read a report of a case of perforation of the bladder, followed by recovery. The case was one requiring great skill in treatment, and the doctor's report was full and satisfactory. Dr. W. also reported an interesting case of uterine hemorrhage, appearing after five and a half months' gestation, caused by detachment of placenta. Premature delivery, favorable to both mother and child. A motion prevailed to file, with a vote of thanks to Dr. Willien for his papers.

In the absence of any report on practical medicine, a random talk was had. Dr. Massie raises the question whether we have any cholagogues.

Drs. Steele, Tenbrook and Chambers think we bleed too seldom. Dr. James Madison would quit the practice if his lancet were taken from him. Dr. Massie would not bleed under any circumstances because it is not fashionable.

A case was introduced by a resident physician for examination by the Society. A committee was appointed to examine the case and report. Committee—Drs. Hays and Willien—report the case staphyloma of the eye, caused by purulent ophthalmia. Case requires very mild treatment—weak solution of sulph. of zinc or nitrate of silver. Let the eye get well, and then excise the staphyloma and put in artificial eye.

In remittent fevers Dr. Steele advocates active catharsis with calomel. Dr. Massie objects to this, because it makes an unusual drain on the portal system. The presence of bile in the dejections is brought about by broken doses of calomel. If the object was to act on the bowels, would give large doses.

Dr. McCord would give an active dose at first, fifteen to thirty grains of calomel to an adult; then four grains of sulph. quinine every four hours.

Dr. A. J. Miller would not give calomel in large doses, if at all. If the tongue is white, don't give calomel, but give alkalies, *quinine as an alterative.*

Dr. McKnown does not believe in scouring out a man every time he gets sick. Gives thirty grains of quinine a day.

Dr. Massie—in every day ague it is questionable whether to give calomel or not—thirty grains of quinine before the next paroxysm.

Dr. McCord does not give large doses of quinine because he does not think such an effect on the nervous system is required ; twelve to fifteen grains is sufficient for him to break up the paroxysm after first moving the bowels freely.

At 8 P. M., Dr. John M. McKnown, of Arcola, Ill., delivered a lengthy and eloquent address. Subject : "Temperance," as viewed from the stand-point of a physician.

On motion, a vote of thanks was tendered the Doctor for his very able address, with a request that a copy be furnished the Society for publication.

After the address the members were furnished complimentary tickets to a supper prepared by the Clark County Medical Society.

The remainder of the evening was delightfully spent in cultivating the spirit of conviviality with the good people of Marshall.

The following toast was read : "The Æsculapian Society, the oldest and most useful medical society in the State," which was responded to by the President in a very happy speech, referring to the origin of the Society, its progress, present prosperity, and its future prospects.

May 28, 7 A. M. The following resolutions were unanimously adopted :

*Resolved*, That this Society hails with pride and admiration the medical educational institutions of our great State of Illinois.

*Resolved*, That while we entertain the most catholic views toward medical colleges of sister States, we know of none possessing superior facilities in the West, both in regard to clinical instruction and able medical teaching.

*Resolved*, That jealousy and rivalry are as reprehensible in medical schools as in individuals, and that we will discountenance and frown down any attempt, on the part of one school, to supercede a sister school in any other way than by genuine merit.

*Resolved*, That we believe the growing interests of the State requires and will sustain two medical schools in Chicago, and that we can conscientiously commend the Rush and Chicago Medical Colleges to such as desire a thorough medical education.

The following are the chairmen of the standing committees : John L. Hays, Surgery ; Mark Rowe, Practical Medicine ; P. H. Barton, Midwifery.

Appointed to write on special subjects for the next meeting : A. J. Miller, Syphilis and Chancroid ; Z. T. Baum, Special Pathology and Therapeutics ; J. L. Hays, the Ear ; J. M. McKown, Is Rheumatism Zymotic ? L. J. Willien, Eliminatives in Disease ; D. O. McCord, Malarial Diseases ; E. B. Cannon, the Eye ; W. S. Martin, Bright's Disease ; A. K. Mosely, Pathology of Remittent Fever.

The following preamble and resolutions were read by Dr. Chambers, chairman of committee, and on motion were unanimously adopted, after appropriate remarks by the chairman of committee and other members of the Society, showing the high estimation of the character and worth of the deceased:

WHEREAS, It has been ordained by the All-Wise Dispenser of life and death that since the last annual meeting of this Society our justly distinguished brother, FLEMING RICH PAYNE, M.D., of Marshall, Illinois, should be removed from his career of usefulness, and intense mental and physical labor, to that state of existence where reigns perpetual peace and repose—where sickness and anguish have no abiding place; and whereas his life was morally blameless, and his connection with this Society shed a lustre upon the organization; therefore

*Resolved*, That by the death of Dr. Payne this Society has lost one of its oldest and best members, the medical literature of the State one of its ablest contributors, the profession at large the teaching and example of a thoroughly scientific physician, and a most worthy Christian gentleman.

*Resolved*, That a copy of this preamble and resolutions be presented to the members of his greatly bereaved family by the Secretary, that they be entered upon the records of the Society, and published in the CHICAGO MEDICAL JOURNAL.

W. M. CHAMBERS,
JOHN TENBROOK,
D. O. MCCORD,
Committee.

Dr. William Massie read his paper on Insanity, which was listened to with profound attention throughout, and was discussed by Drs. Chambers, Steele and Rev. Mr. Pilcher, all conceding the ability and research exhibited by the writer.

It would be difficult to give a synopsis of this paper without reproducing the whole, but the following is a summary of its conclusions:

1. There are degrees of responsibility in the insane.

2. Insanity does not necessarily acquit of responsibility, though it should be regarded as a mitigating circumstance.

3. No unexceptionable test of responsibility can be laid down, therefore each case should be decided for and by itself without reference to precedent.

4. The jury *alone* should determine the degree of responsibility of each concrete case upon testimony.

5. Three questions arise in every alleged case of insanity:

*First.* Is the accused insane?

*Second.* If so, is the alleged crime the legitimate product or out-come of the insanity?

*Third.* To what degree is the accused responsible for his act?

6. The medical expert *alone* is competent to testify on the first question.

7. Moral insanity, without impairment of the intellectual faculties, is a species of depravity, and should not acquit of crime.

8. Drunkenness is insanity, and should sometimes acquit of crime; at other times should be regarded as a mitigating circumstance.

9. Voluntary drunkenness is therefore itself a crime.

Drs. Ragan, Massie and Steele were appointed a committee to correspond with medical journals of Chicago in reference to the publication of valuable papers and transactions of the annual and semi-annual meetings of the Society.

A vote of thanks was tendered to the Clark County Medical Society, the citizens and Methodist Church of Marshall, for their courtesy, liberality and elegant entertainment.

Many other things of importance were brought before the meeting, but not of so general a character as to be published.

Neoga, Illinois, was selected as the place of holding the next annual meeting, which will be the 18th and 19th of November, 1874.

